# Harmful Gas Recognition Exploiting a CTL Sensor Array

**DOI:** 10.3390/s131013509

**Published:** 2013-10-09

**Authors:** Qihui Wang, Lijun Xie, Bo Zhu, Yao Zheng, Shihua Cao

**Affiliations:** 1 School of Aeronautics and Astronautics, Zhejiang University, Hangzhou 310027, China; E-Mails: zdxlj@zju.edu.cn (L.X.); zhubo@zju.edu.cn (B.Z.); yao.zheng@zju.edu.cn (Y.Z.); 2 Qianjiang College, Hangzhou Normal University, Hangzhou 310012, China; E-Mail: caoshihua@126.com

**Keywords:** CTL, sensor array, pattern recognition

## Abstract

In this paper, a novel cataluminescence (CTL)-based sensor array consisting of nine types of catalytic materials is developed for the recognition of several harmful gases, namely carbon monoxide, acetone, chloroform and toluene. First, the experimental setup is constructed by using sensing nanomaterials, a heating plate, a pneumatic pump, a gas flow meter, a digital temperature device, a camera and a BPCL Ultra Weak Chemiluminescence Analyzer. Then, unique CTL patterns for the four types of harmful gas are obtained from the sensor array. The harmful gases are successful recognized by the PCA method. The optimal conditions are also investigated. Finally, experimental results show high sensitivity, long-term stability and good linearity of the sensor array, which combined with simplicity, make our system a promising application in this field.

## 1. Introduction

Harmful gases can affect human health. For example, carbon monoxide is widely used in industry, but due to its odorlessness, anesthetic effect and flammability, carbon monoxide can lead to death if people are exposed to it for even a brief time [1]. It may damage the nervous system and cause feeling, response, comprehension, and memory dysfunctions by accessing the blood circulation through the respiratory tract [2]. Some other harmful gases, such as acetone, chloroform and toluene are usually important auxiliary materials utilized by drug traffickers even though they are not drugs in their own right. As the international security issue is becoming more and more serious, fast detection of toxic gas is required [3]. Automobiles have become indispensable transport tools, providing great convenience to human beings. However, automobile exhaust may corrode buildings, damage human health and destroy the city landscape. Nitrogen oxides in automobile exhaust can produce strong stimulation and damage to the lungs and result in pulmonary lesions. When the concentration of nitrogen oxides reaches 10–20 ppm, it may stimulate the nasal mucosa, pharynx, trachea and cornea, whereas the concentration is more than 500 ppm, it may make person suffer emphysema and even death [4]. Obviously, sensitive, stable and portable instruments for detecting harmful gases are necessary.

Gas sensors have been widely used in gas control, public safety and environmental protection [5–7]. In recent years, gas sensors using optical methods have been a very important research hotspot. Researchers have made great progress in instrumentation design and construction, sensing material development and establishment of luminescence mechanisms [8,9]. Cataluminescence (CTL) was first described by Breysse and his co-workers who studied a catalytic oxidation reaction of CO on the surface of heated solid materials in an atmosphere containing oxygen [10]. Since then, persistent efforts have been made to develop CTL analytical methods for the detection of harmful gases such as benzene [11], acetaldehyde [12], formaldehyde [13] and benzaldehyde [14].

Recently, CTL sensor arrays have attracted great research interest. The major advantage of a CTL sensor array is the ability to collect many kinds of information quickly, such as luminescence intensity, luminescence lifetime, wavelength and spectral shape [15]. Furthermore, the sensing materials of CTL-based sensor arrays are solid catalysts, so consumption of these materials in the sensing reactions is very low [16], which means that these new sensor arrays possesses long-term stability. Therefore, CTL provides a novel sensing strategy for the detection and identification of harmful gases [17]. In summary, CTL-based sensors show significant advantages over traditional sensors, such as stability, durable intensity, high signal/noise (S/N) ratio and rapid response, there is also nearly no consumption of sensing materials during the detection process [18].

Typically, a CTL sensor image can be used for the detection of a harmful gas, but in many application areas, in order to perfectly and accurately reflect the results, often requires the simultaneous measurement of multiple gases. Researchers have shown increasing interest in developing universal gas sensing systems, such as arrays or column gas sensors. These sensor systems is often named “electronic noses”. An electronic nose usually consists of a cross-sensitivity sensor array and related data processing modules. With an appropriate pattern recognition algorithm, the sensor system has ability to smell and identify several harmful gases in a complex environment. This method is mainly used to solve multiple odor detection problems. As an electronic nose array sensor is composed of different sensing materials, the number of configurations is also different. In 1995, Nakagawa realized this method, as since the catalytic luminescence process is a heterogeneous catalyst reaction, a variety of interactions exist between units at the same time [19]. The reactions can provide a lot of information for detection and recognition of harmful gases. He proposed a multi-sensor system to identify a variety of gases, at the same time, an evaluation of catalytic activity was presented. In the 2000s, Zhang and his team reported a lot of research on CTL sensor arrays, and their studies further developed this multi-sensor system. They first reported an initial proof-of-principle work on a nanomaterial-based optical chemosensor array in a communication [20], then a cross-reactive CTL sensor array based on catalytic nanomaterials was constructed for the discrimination and identification of flavors in cigarettes [21]. They also proposed cataluminescence-based array imaging as a high-throughput screening technique in the combinatorial discovery of active catalysts for CO oxidation [22].

In our study, we tried to use nine types of catalytic luminescent materials to simultaneously detect harmful gases including carbon monoxide, acetone, chloroform and toluene. These four kinds of harmful gas were detected and discriminated based on their distinct CTL patterns. The CTL patterns were obtained by the sensor array and an imaging method for these harmful gases. Moreover, fast evaluations of carbon monoxide, acetone, chloroform and toluene were carried out at an optimal working temperature. The principle component analysis (PCA) method was used in order to illustrate the selectivity of this sensor array. The analytical characteristics and temperature effect of the CTL-based array were investigated. As a result, the excellent linearity and stability indicated the feasibility of using this array for the determination of carbon monoxide, acetone, chloroform and toluene. The experimental data verified the sensitivity of our sensor imaging method. Finally, our paper conceived the possibility to identify more harmful gases by increasing the types of sensing material used (for example, by adding Y_2_O_3_-based sensors) and improving the image processing technology (by using a data fusion method for the visible and IR image).

## 2. Experiments

### 2.1. Sensor Device, Sensing Materials and System Architecture

**Figure 1.**
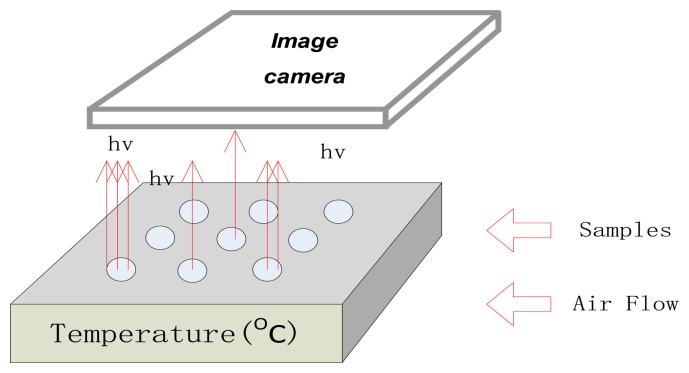
Brief structure of the CTL sensor device.

**Figure 2.**
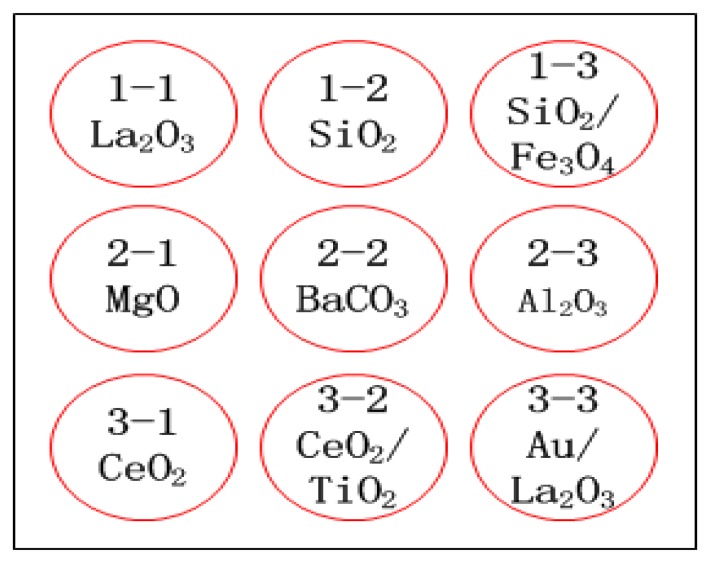
Arrangement of material spots on the sensor array.

A diagram of the sensor device is presented in Figure 1. Sensing nanomaterials, including nano-sized oxides (La_2_O_3_, SiO_2_, MgO, Al_2_O_3_, CeO_2_), decorated nanoparticles (SiO_2_/Fe_3_O_4_, CeO_2_/TiO_2_, Au/La_2_O_3_) and carbonates (BaCO_3_) were synthesized. The synthesis procedures of these nanomaterials had been investigated and described in previously published papers, such as [8,12,17,18,21,23–26]. As shown in Figure 2, the sensing materials were neatly embedded into the surface of a heating plate to form a 3 × 3 array (about 0.2 mm in thickness and 8 mm in diameter for each sensing element). Air flow was supplied by a pneumatic pump.

**Figure 3.**
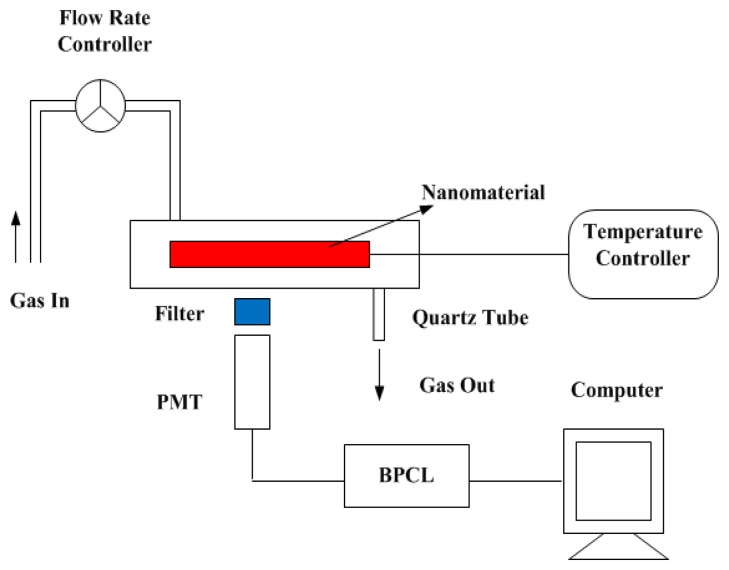
Schematic diagram of the CTL sensor array system.

In our experiments, a precise flow meter was employed for measurement of the gas flow rate. The temperature of the heating plate was controlled by a digital temperature device. The final CTL patterns were recorded by a camera near the heating plate. All chemicals, including carbon monoxide (purity ≥99.0%), acetone (purity ≥99.5%), chloroform (purity ≥99.7%) and toluene (purity ≥99.5%) used in the experiments had high purity analytical grade. Thus they could be used in our experiments without any further purification. The reagents used in our experiments were purchased from the Shanghai Chemical Reagents Company (Shanghai, China). The apparatus used for detecting and processing the weak chemiluminescence (CL) signals was a BPCL Ultra Weak Chemiluminescence Analyzer, which was produced by the Biophysics Institute of the Chinese Academy of Sciences (Beijing, China). The gas entered the reactor with a flow rate of 200 mL/min. A schematic diagram of the CTL sensor array system is shown in Figure 3.

### 2.2. Image Processing

A typical image processing application system includes a light source, image capturing system, digital image processing module, intelligent decision-making module and a mechanical control execution module. A camera or another image capturing device converts optical signals into digital signals, then these will be sent to a dedicated image processing system. According to the distribution of pixels, brightness and color information, a series of algorithms will be executed to extract the features. In this section, we talk about the image acquisition, image fusion and pattern recognition of our system.

#### 2.2.1. Image Acquisition

Image acquisition is actually a photoelectric conversion process. It is responsible for capturing the real image of the object, the analog image signal is converted to digital signal, and ultimately into computer data. It provides on-site real-time data for further processing, and directly affects the image quality and image processing effect. Our study focused on the CTL-based imaging method for harmful gases, and aside from exploiting BPCL, we also designed other image acquisition devices according to the characteristics of different applications. There are many advantages of using CTL-based imaging methods for the detection and recognition of harmful gases. The major advantage is the ability to collect CTL emissions from various catalysts simultaneously. In order to test and verify the system, four kinds of harmful gas were examined in our study, including carbon monoxide, acetone, chloroform and toluene.

#### 2.2.2. Image Fusion

A multi-sensor image fusion system has a broad spatial and temporal coverage area, excellent target resolution capability, good fault tolerance ability, excellent reconstruction and system detection performance and high measurement dimensions. They were first used in military target detection and recognition. At present, multi-sensor image fusion technology has a variety of applications in air traffic control, surveillance, robot vision, harbor high speed transport driving, natural resources, weather forecasting and remote testing. Therefore, multi-sensor image fusion technology has developed rapidly, and the aim is to collect more information from a number of single sensors, and supplementary information could be merged into a new data set [27]. Studies on the characteristics of human vision found that human vision is quite sensitive to color, and while there are only a few dozen gray-levels which eyes can distinguish, color resolution can reach hundreds or even thousands of colors. Compared to the gray information, the human eye can more quickly and accurately identify color-coded information. In 1992, Walraven and Lucassen fused two images of different frequency with the pseudo color mapping method. For the fused image, they use the observer performance evaluation. The results showed that the pseudo-color coding information improved the recognition rate by 30%, and the recognition error rate is reduced by 60% [28].

In our experiments, the characteristics of the CTL-based array performance for carbon monoxide, acetone, chloroform and toluene sensing were investigated under optimal conditions. Considering the fact that the fused images were not natural, a revised region-based color mapping method was employed to render the multi-sensor images [29]. Experiments showed that the result images contained more details, so the harmful gases could be recognized more easily.

#### 2.2.3. Pattern Recognition

PCA was used to classify the harmful gases and the results of nine sensor units for four chemicals were obtained. During our experiments, carbon monoxide, acetone, chloroform and toluene underwent five repeated tests under optimal conditions. Two PCs accounted for 97.60% of the total variance (PC1: 70.04%, PC2: 27.56%). In the test process, the acquired images were first converted by the image digitizer and input into an appropriate buffer. Then, according to the operator's instructions, the computer calls the image processing program in the library. After image processing, the computer generated output images pixel by pixel, they were transmitted into a cache or another actuating mechanism. Finally, the processing results can be recorded by the recording equipment.

## 3. Results and Discussion

### 3.1. Sensitivity

**Figure 4.**
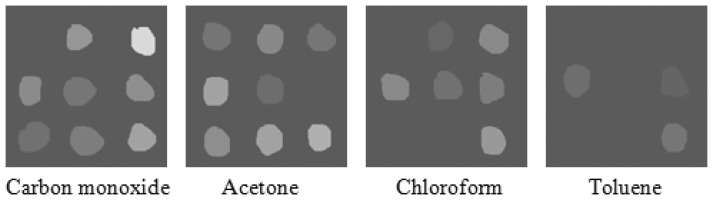
CTL sensor images of carbon monoxide, acetone, chloroform and toluene. Concentration of all compounds: 1000 ppm, working temperature: 240 °C, carrier gas flow rate: 200 mL/min.

**Figure 5.**
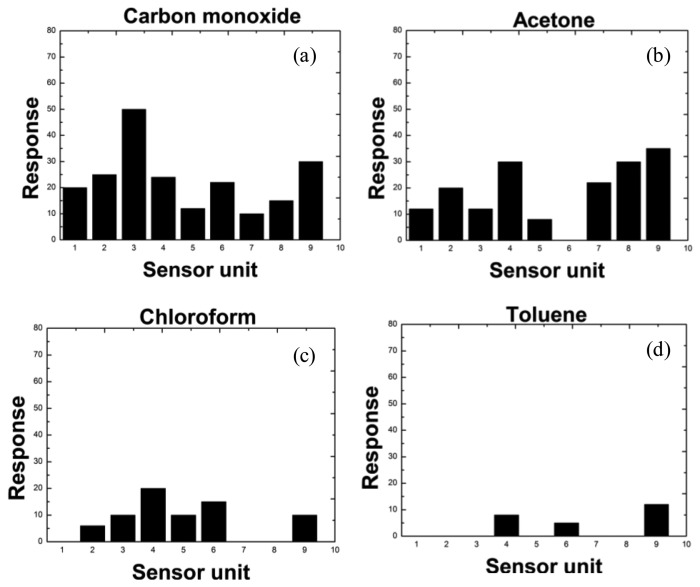
Brightness histograms obtained from CTL patterns of carbon monoxide, acetone, chloroform and toluene on each sensing element of the array. Concentration of all compounds: 1000 ppm, working temperature: 240 °C, carrier gas flow rate: 200 mL/min.

To evaluate the correlation between catalytic activity and CTL response, nine types of catalyst (see Figure 2) with different activities for carbon monoxide, acetone, chloroform and toluene were applied. Figure 4 demonstrates the unique response patterns of these compounds, which were acquired from the sensor system. The mean responses of the four kinds of gas on different sensing elements are shown in Figure 5. From the figures, the use of the fabricated cross-sensitivity sensor array was confirmed to feasible for detecting and recognizing these harmful gases.

**Figure 6.**
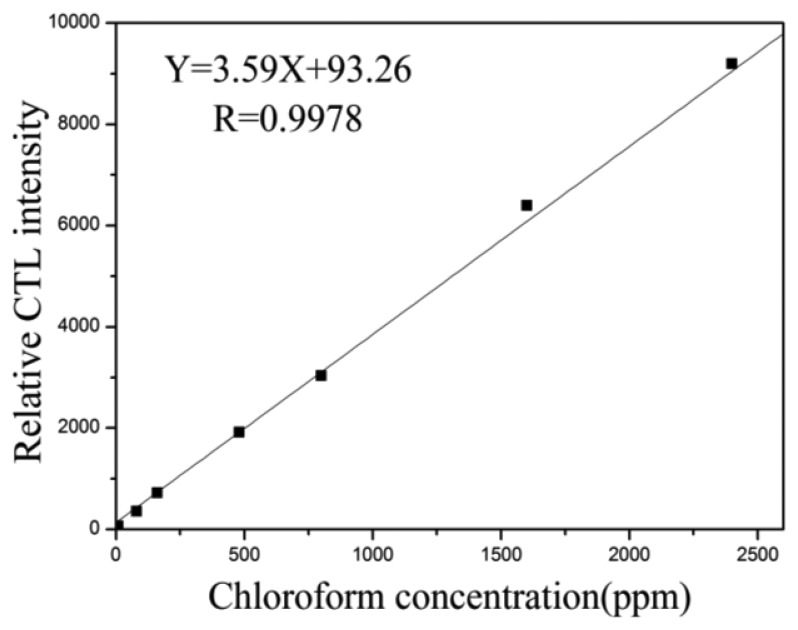
The calibration curve for chloroform, working temperature: 240 °C, carrier gas flow rate: 200 mL/min.

The CTL intensities change with different metal catalysts, total metal loadings and ratio of bimetallic catalysts. Taking the catalysts at a metal loading of 1.0% as examples, on average, the spot of SiO_2_/Fe_3_O_4_ (1/3) has the highest CTL intensity, the spot of La_2_O_3_ (1/1) gives the weaker intensity, whereas the weakest one is BaCO_3_ (2/2). The conversion coefficient of carbon monoxide, acetone, chloroform and toluene for these three catalysts is 0.99. In conclusion, a good correlation between carbon monoxide, acetone, chloroform and toluene conversions can be obtained, so qualitative results for the catalytic sensitivities of those sensing materials for carbon monoxide, acetone, chloroform and toluene can be acquired from the CTL-based array images. In addition, the sensor array exhibits sensitive and stable CTL responses to the four compounds, and the relative CTL intensity increases with their concentrations. For example, responses of chloroform on the sensor utilizing the Au/La_2_O_3_ nanomaterial are shown in Figure 6. The detection limit is 5 ppm and the linear range is 10–2500 ppm (*R* = 0.9978). The quantitative analysis of this system indicates that it can be applied to quantify the concentrations of harmful gases according to the reaction intensity.

### 3.2. Wavelength

**Figure 7.**
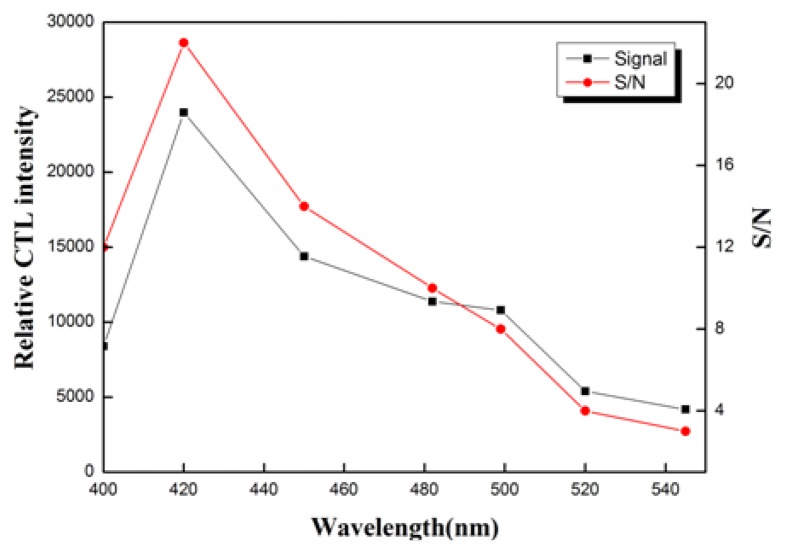
Mean CTL intensity and S/N ratio on Au/La_2_O_3_ nanomaterial.

The relation between CTL spectra wavelength, mean CTL intensity and S/N ratio for the four types of gases on the Au/La_2_O_3_ nanomaterial is shown in Figure 7. Because of the high working temperature, the incandescent radiation noise signals at longer wavelength are higher than those at shorter wavelength. Figure 7 shows that 420 nm is an approximately optimal wavelength, so it is selected for the quantitative detection because of the high S/N ratio.

### 3.3. Temperature

**Figure 8.**
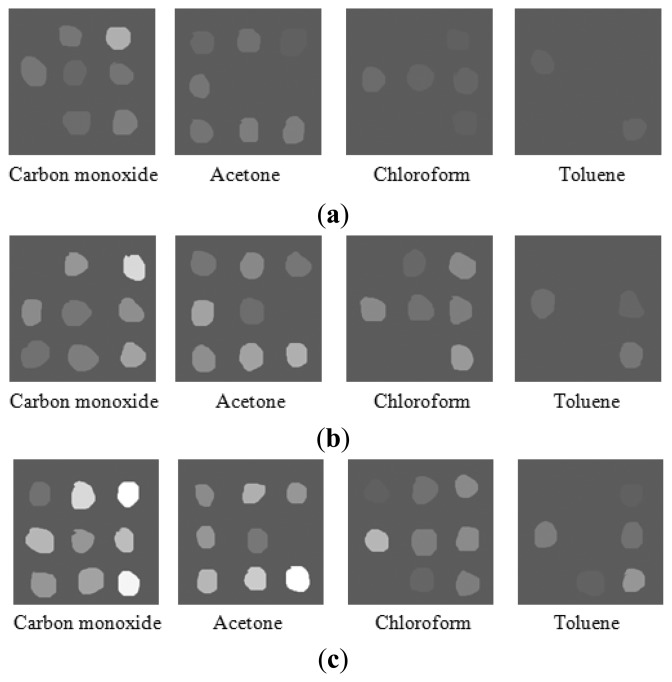
The patterns of carbon monoxide, acetone, chloroform and toluene at different working temperatures. Concentration: 1000 ppm; carrier gas flow rate: 200 mL/min. (**a**) Working temperature: 180 °C; (**b**) Working temperature: 240 °C; (**c**) Working temperature: 280 °C.

In optimizing the CTL-based sensor array, temperature is an important factor which must be taken into account, because the catalytic reaction is temperature dependent. Different temperatures may lead to different luminescence efficiencies during the course of discriminating one compound from another, which might have similar chemical properties. As shown in Figure 8, the experiments were operated at different working temperatures and different image patterns of the sensor array for discrimination of carbon monoxide, acetone, chloroform and toluene were investigated.

In regard to chloroform and toluene, patterns with a relatively weak spot brightness were obtained at 180 °C. When the temperature was increased to 240 °C and 280 °C, the brightness of the spots on the patterns was sufficient to differentiate between these four compounds. In our research, 240 °C was chosen as the optimal working temperature for detection and recognition of carbon monoxide, acetone, chloroform and toluene gases. The results confirm that different patterns can be obtained at different working temperatures which may contribute to distinguishing harmful gases.

### 3.4. Stability

In our work, the stability of a single sensor unit and the whole array were all investigated. We found that the CTL-based sensor array showed excellent stability and durability toward carbon monoxide, acetone, chloroform and toluene. The results were obtained by testing the four kinds of common possible harmful compounds under the optimal conditions. For example, the CTL intensities of Au/La_2_O_3_ sensor unit were collected every 2 h. The single sensor exhibited good stability and durability when we continuously injected 1000 ppm carbon monoxide for 100 h. The sensor response intensity variation was limited to ±10%. As to the sensor array, the CTL patterns of acetone during 0–100 h was investigated. Most sensor units show no significant differences in CTL response. However, the response intensities on SiO_2_/Fe_3_O_4_ (1/3) and BaCO_3_ (2/2) suffered a slight decrease after 100 h.

### 3.5. Selectivity

**Figure 9.**
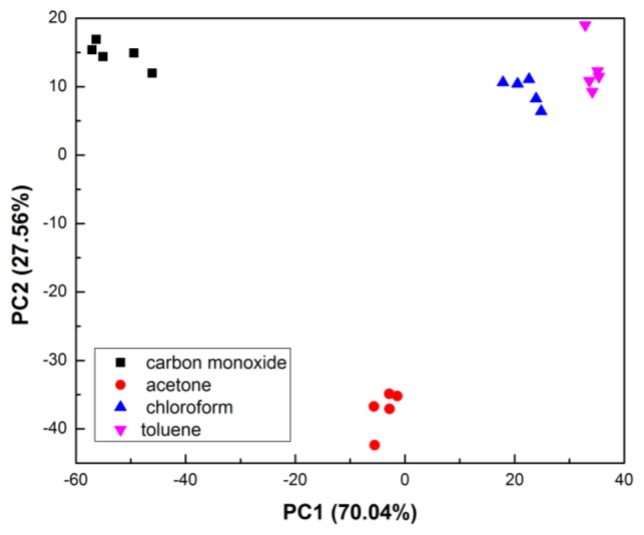
PCA score plots of carbon monoxide, acetone, chloroform and toluene.

Selectivity is the ability of the system to distinguish one analyte from another, which is an important criterion in selecting a sensor array. In Figure 9, the PCA results demonstrated that different point sets acquired from the carbon monoxide, acetone, chloroform and toluene patterns were well classified. The two dimensional PCA scoreboard showed clear coding of 20 injections, and the recognition rate was high. These pattern recognition results of the harmful gases illustrate the excellent selectivity and repeatability of this sensor array.

## 4. Conclusions

Firstly, a novel CTL-based sensor array consisting of nine types of catalytic materials was developed for the detection and recognition of harmful gases including carbon monoxide, acetone, chloroform and toluene. The sensing materials were composed of nanosized metal oxides (La_2_O_3_, SiO_2_, MgO, Al_2_O_3_, CeO_2_), decorated nanoparticles (SiO_2_/Fe_3_O_4_, CeO_2_/TiO_2_, Au/La_2_O_3_) and carbonates (BaCO_3_). They were selected and carefully synthesized. Secondly, distinct CTL patterns for the four types of harmful gas were obtained. Therefore, the harmful gases could be successfully recognized by the PCA method. Thirdly, the optimal conditions were determined: 420 nm was taken as the approximate optimal wavelength, 240 °C was chosen as the optimal working temperature, and the gas flow rate was 200 mL/min. In our experiments, characteristics of the system were investigated under optimal conditions. Fourthly, experimental results showed high sensitivity and long-term stability of the array, combined with simplicity, making our system exhibited a promising application. Finally, a good linear correlation between gas concentration and CTL intensity was verified, which indicated that the CTL intensity can be used in the evaluation of harmful gas concentrations.

## 5. Outlook

Our approach has good potential to develop a CTL sensor array for analysis of other harmful compounds. There are many efficient sensing materials have been developed for gas sensors, such as Au/ZnO, SnO_2_, Y_2_O_3_ and Mn_3_O_4_, that could be integrated into our system. In addition, improving the image fusion method maybe contribute to develop an improved CTL sensor array, and we will attempt to implement a color image fusion algorithm based on the Curvelet transform [30], which is supposed to help the observer interpret the image more intuitively.
